# Is dexmedetomidine superior to non-dexmedetomidine sedatives (particularly propofol) for sedation in critically ill patients with septic shock? A systematic review and meta-analysis of randomized controlled trials

**DOI:** 10.3389/fmed.2025.1646256

**Published:** 2025-10-09

**Authors:** Xinjing Gao, Zhaoting Li, Zhibo Li, Yingzhi Qin, Jie Ren, Kai Zhang, Wenjiao Wang

**Affiliations:** ^1^Department of Critical Care Medicine, The Third Central Hospital of Tianjin, Tianjin, China; ^2^Department of Critical Care Medicine, Tianjin University, Affiliated Center Hospital, Tianjin University, Tianjin, China; ^3^Tianjin Key Laboratory of Extracorporeal Life Support for Critical Diseases, The Third Central Hospital of Tianjin, Tianjin, China; ^4^Artificial Cell Engineering Technology Research Center, The Third Central Hospital of Tianjin, Tianjin, China; ^5^The Third Central Clinical College, Tianjin Medical University, Tianjin, China

**Keywords:** septic shock, sedation, dexmedetomidine (DEX), non-dexmedetomidine (non-DEX), propofol (PROP)

## Abstract

**Background:**

Dexmedetomidine (DEX) and propofol (PROP) are both recommended as first-line short-acting sedative-analgesic agents for sepsis patients. However, existing studies have reported inconsistent clinical outcomes potentially attributable to their distinct hemodynamic profiles. The aim of our study was to systematically evaluate the comparative clinical efficacy and safety of DEX vs. non-Dexmedetomidine sedatives (particularly Propofol) in patients with septic shock.

**Methods:**

The study protocol was prospectively registered on PROSPERO (CRD42024626139). Randomized controlled trials (RCTs) meeting eligibility criteria were systematically searched up to December 2024. Statistical analyses were performed using RevMan 5.4, and trial sequential analysis (TSA) was employed to determine the required sample size.

**Results:**

17 RCTs were enrolled with 1,422 patients. Compared with non-DEX group, DEX group presented significantly reduced 28-day mortality (odds ratio [OR] 0.68, 95% CI 0.49–0.94, *p* = 0.02), lower IL-6 (mean difference [MD] −3.11 ng/L, 95% CI −5.32 to −0.90, *p* = 0.006) and TNF-*α* (MD −0.21 ng/L, 95% CI −0.30 to −0.12, *p* < 0.001). Importantly, the incidence of adverse effects did not increase compared to non-DEX groups, as evidenced by delirium (OR 0.82, 95% CI 0.34 to 1.97, *p* = 0.66), bradycardia (OR 1.36, 95% CI 0.66 to 2.78, *p* = 0.40), and hypotension (OR 1.38, 95% CI 0.59 to 3.19, *p* = 0.46). In the subgroup analysis, PROP showed no significant differences over DEX for key clinical outcomes, including: 28-day mortality and duration of invasive mechanical ventilation (IMV), length of stay in Intensive Care Unit (ICU LOS), etc. Regrettably, existing RCTs lacked sufficient data regarding inflammatory biomarkers and adverse event profiles above in direct comparisons between DEX and PROP. TSA on 28-day mortality between DEX and PROP indicated that a minimum of 1,269 additional participants would have required to achieve conclusive evidence (*α* = 0.10; *β* = 0.30; relative risk reduction [RRR] = 12.5%).

**Conclusion:**

DEX demonstrated superiority over non-DEX sedatives in critically ill patients with septic shock without increasing hemodynamic adverse events. However, current evidence showed no significant differences between DEX and PROP, warranting further high-quality RCTs for definitive conclusions.

## 1 Background

Sedation is clinically indicated for patients in ICU (1), and dexmedetomidine (DEX) and propofol (PROP) are firstly recommended by guidelines to achieve desired sedation while reducing opioid consumption (2). In patients with septic shock, who exhibit profound circulatory failure and consequent cellular metabolic abnormalities than those with sepsis alone, sedatives can not only provide analgesia and attenuate the stress response but also reduce metabolic demand, improve ventilator synchrony, alleviate anxiety and discomfort, and even confer potential organ-protective effects (3). However, due to the therapeutic challenges in avoiding aggravated hypotension and regulating organ function of septic shock patients (4), clinicians face a difficult dilemma when choosing between DEX and PROP.

DEX was previously considered superior than others for sepsis, but the current opinions on its efficacy remained divided. The results of a meta-analysis including 5 trials (540 patients with septic shock) showed that DEX significantly reduced the sequential organ failure assessment (SOFA) score and the duration of invasive mechanical ventilation (IMV) support in patients with septic shock (5). However, recent studies have also demonstrated that DEX did not show a significant advantage over other sedation strategies in improving clinical outcomes (6), even though it has shown that DEX does not worsen the hemodynamic parameters in patients with septic shock (7). It may be related to the exacerbation of sepsis-induced cardiac inflammation and myocardial dysfunction, at least in part through the activation of cardiac endothelial *α*-adrenergic receptors following DEX treatment (8).

Moreover, there was no consensus regarding DEX against PROP yet. Two randomized controlled trials (RCTs) reported that DEX was not inferior to PROP in ICU patients receiving prolonged IMV (9). And, a meta-analysis reported that DEX was associated with lower delirium incidence among older adults in ICU with no significant increase in adverse events (10). But, another meta-analysis showed that DEX significantly increased the risk of bradycardia compared to PROP (11). Animal research also indicated that PROP and DEX had a differential impact on preload dependence in endotoxin models (12). In the endotoxin shock model after fluid resuscitation during norepinephrine infusion, PROP was more effective than DEX in increasing pulse pressure variation (PPV), which indicated a good response to fluid therapy in patients, and suggested that PROP was more conducive to improving cardiac output and tissue perfusion compared to DEX. In contrast, DEX decreased heart contractility and increased vascular resistance at the highest dose. Although there were no more studies on this mechanism, it did not prevent the speculation that PROP may have a better advantage than DEX in sedation in patients with septic shock.

The aim of our study is to clarify that whether DEX, compared to other sedation measures (particularly PROP), could improve clinical outcomes for patients with septic shock, while maintaining well safety.

## 2 Methods

### 2.1 Literature search and study selection

This systematic review adhered to the Preferred Reporting Items for Systematic Reviews and Meta-Analyses (PRISMA) guidelines (Supplementary material 1) (13). The study protocol was prospectively registered in PROSPERO (CRD42024626139).

We conducted comprehensive literature searches in PubMed, Embase, China National Knowledge Infrastructure (CNKI), China science and technology journal (VIP) database, and Cochrane Library for RCTs published in English or Chinese up to December 2024. The search strategy incorporated broad terms related to “septic shock” and “dexmedetomidine (DEX)” to maximize sensitivity. The full search syntax for each database was detailed in Supplementary material 2 to ensure transparency and reproducibility.

**Figure 1:**
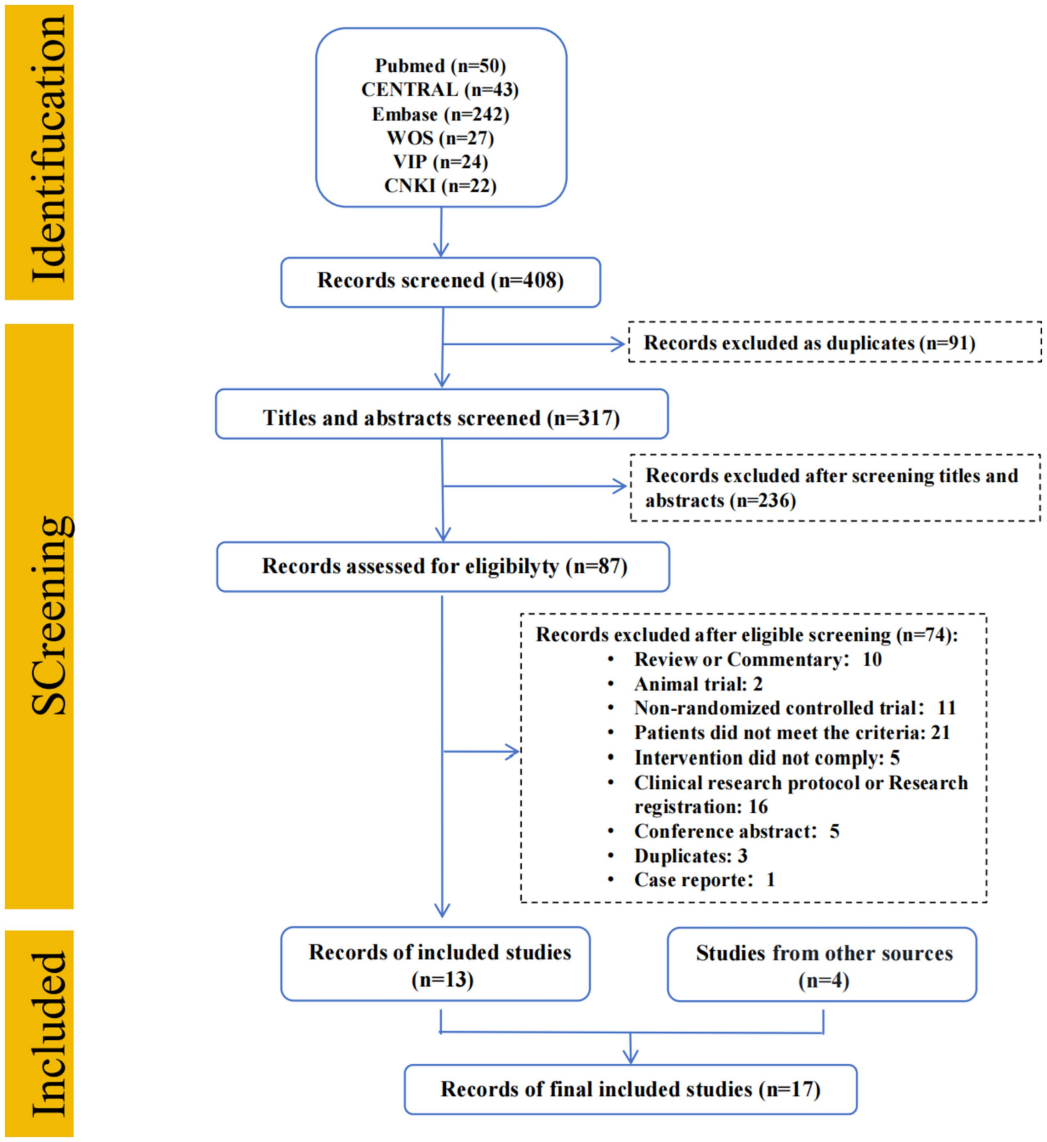
Flow diagram. CENTRAL, Cochrane central register of controlled trials; EMBASE, Excerpta medica database; WOS, Web of science; VIP, China science and technology journal database; CNKI, China national knowledge infrastructure.

To minimize publication bias, we supplemented the electronic search with manual screening of reference lists from relevant articles and reviews. And, the screening workflow of study selection was presented in Figure 1.

### 2.2 Inclusion and exclusion criteria

Studies were eligible if they met all the following criteria: ① Population: Adult patients (≥18 years) with septic shock, diagnosed according to internationally recognized criteria (e.g., Sepsis-3 or prior consensus definitions); ② Intervention: Intravenous DEX, irrespective of dose, timing, or duration of administration; ③ Comparison: PROP, other intravenous sedatives, or placebo (any dose or regimen). ④ Study design: RCTs.

Studies were excluded if they met any of the following: ① Population: Non-human studies (animal or *in vitro* experiments) or pediatric populations; ② Disease definition: Diagnostic criteria inconsistent with septic shock, or failed to distinguish between septic shock and uncomplicated sepsis; ③ Study design: Non-RCTs (e.g., observational studies, case reports, reviews); ④ Duplication: Redundant publications or overlapping datasets; ⑤ Language: Non-English or non-Chinese publications; ⑥ Data availability: Unretrievable full text or incomplete/unusable data.

### 2.3 Data extraction

The data necessary for the following analysis were extracted and entered into Table 1, including: basic information of the study (first author, year of publication, etc.), characteristics of the study subjects (sample size, gender composition, average age, disease severity), specific interventions for the experimental (DEX) and control groups (non-DEX or PROP) and methodological quality indicators.

**Table 1:** Detailed information of included studies.

Study	Research type	Blind method	Sample size	Male	Age	APACHE II score	SOFA score	Intervention	Sedation goals
Chen L (29)	Single-center RCT	NA	EG: 40 CG: 40	NA	NA	NA	NA	EG: DEX CG: Usual care	/
Cioccari L (30)	International RCT	Open label	EG: 44 CG: 39	EG: 29 CG: 28	EG: 67.7 ± 12.4 CG: 62.9 ± 16.8	EG: 24.9 ± 6.7 CG: 25.3 ± 7.0	EG: 6 [5–10] CG: 9 [5–14]	EG: DEX CG: PROP/Midazolam	RASS: −2 to 1
Elayashy M (31)	Single-center RCT	Double blind	EG:12 CG:12	EG: 6 CG: 6	EG: 55.4 ± 10.2 CG: 50.8 ± 14	EG: 11.1 ± 4.7 CG: 11.3 ± 4.6	EG: 6 [5–7] CG: 6 [5–7.8]	EG: DEX CG: Midazolam	RASS: −3 to −1
Ezz Al-Regal et al. (6)	Single-center RCT	Open label	EG: 45 CG: 45	EG: 24 CG: 24	EG: 59 ± 16.6 CG: 61 ± 14	EG: 28.8 ± 5.2 CG: 27.8 ± 5.1	EG: 11 [10–12] CG: 11 [10–11.5]	EG: DEX CG: Midazolam/Fentanyl	RASS: −4 to 1
Guo F (32)	Single-center RCT	NA	EG:14 CG:16	NA	EG: 54.9 ± 20.7 CG: 58.2 ± 19.1	EG: 24.1 ± 4.0 CG: 22.5 ± 4.5	NA	EG: DEX CG: Midazolam/PROP	RASS: −1 to −2
Kadoi Y (33)	Single-center RCT	Double blind	EG: 10 CG: 10	NA	EG: 66 ± 7 CG: 65 ± 8	EG: 37 ± 5 CG: 38 ± 4	NA	EG: DEX CG: PROP	RSS: 4
Lan Y (34)	Single-center RCT	NA	EG: 41 CG: 41	EG: 24 CG: 21	EG: 40.2 ± 5.3 CG: 42.5 ± 9.2	EG: 22 ± 4.4 CG: 23 ± 5.1	NA	EG: DEX CG: Midazolam	Ramsay: 2 to 4
Liu J (35)	Single-center RCT	Double blind	EG:100 CG:100	EG: 57 CG: 58	EG: 57 [31–66] CG: 54 [35–71]	EG: 29 [26–37] CG: 29 [22–36]	EG: 10 [8–13] CG: 11 [8–12]	EG: DEX CG: PROP	RASS: −2 to 0
Memiş D (36)	Single-center RCT	Open label	EG: 20 CG: 20	EG: 14 CG: 13	EG: 60 [31–80] CG: 54 [25–78]	EG: 22 ± 5 CG: 20 ± 8	EG: 4.5 ± 2.8 CG: 4.0 ± 2.9	EG: DEX CG: PROP	RSS: ≤2
Miyamoto K (37)	Multicenter RCT	Double blind	EG: 60 CG: 51	NA	EG: 70.0 ± 14.3 CG: 72.1 ± 12.3	EG: 23 [19–29] CG: 27 [20–32]	EG: 10 [8–12] CG: 11 [8–12]	EG: DEX CG: Placebo	RASS: −2 to 0
Mokhlesian M (7)	Single-center RCT	Double blind	EG: 24 CG: 24	EG: 15 CG: 10	EG: 59.6 ± 18.3 CG: 61.7 ± 6.4	EG: 20.2 ± 5 CG: 19.9 ± 4.7	EG: 8.9 ± 2.3 CG: 9.4 ± 2.5	EG: DEX CG: Midazolam/Morphine	RASS: −2
Nakashima T (38)	Multicenter RCT	Double blind	EG: 54 CG: 50	EG: 30 CG: 33	EG: 70.7 ± 15.1 CG: 71.4 ± 13.2	EG: 29 [25–31] CG: 30 [25–33]	EG: 9 [7–11] CG: 11 [9–13]	EG: DEX CG: Placebo/Midazolam	RASS: −2 to 0
Ohta Y (39)	Multicenter RCT	Double blind	EG: 100 CG: 101	EG: 63 CG: 64	EG: 68 ± 14.9 CG: 69 ± 13.6	EG: 23 [18–29] CG: 22 [16–29.5]	EG: 8 [6–11] CG: 9 [5–11]	EG: DEX CG: Placebo	RASS: 0 to −2
Tasdogan M (40)	Single-center RCT	Double blind	EG: 20 CG: 20	NA	EG: 58 [21–78] CG: 50 [19–74]	EG: 18 ± 4 CG: 19 ± 5	EG: 4.2 ± 1.8 CG: 4.0 ± 2.5	EG: DEX CG: PROP	Ramsay: ≤ 2 BPS: ≤5
Wang L (41)	Single-center RCT	NA	EG: 15 CG: 15	EG: 7 CG: 6	EG: 66 ± 19 CG: 64 ± 16	EG: 23 ± 3 CG: 24 ± 3	NA	EG: DEX CG: Midazolam	RASS: −2
Wei G (42)	Single-center RCT	NA	EG: 60 CG: 59	EG: 33 CG: 30	EG: 43.5 ± 7. 9 CG: 45.2 ± 8. 4	EG: 26.4 ± 5.2 CG: 25.1 ± 5.9	EG: 12.4 ± 2.8 CG: 11.8 ± 2.5	EG: DEX CG: PROP	/
Zhang C (43)	Single-center RCT	NA	EG: 60 CG: 60	NA	EG: 48.4 ± 8.9 CG: 48.1 ± 9.4	EG: 11.7 ± 3.5 CG: 11.5 ± 3.6	NA	EG: DEX CG: Midazolam	Richmond: −2 to 1

RCT, Randomized Controlled Trial; EG, Dexmedetomidine Group; CG, Control Group; NA, Not Available; DEX, Dexmedetomidine; PROP, Propofol.

Meanwhile, prognostic indicators including primary (28-day mortality) and secondary outcomes [duration of IMV, length of stay in intensive care unit (ICU LOS)], adverse events (e.g., delirium, bradycardia, hypotension), and inflammatory markers [interleukin-6 (IL-6), tumor necrosis factor-alpha (TNF-*α*)] were also extracted.

To ensure accuracy and minimize bias, data extraction was performed independently by two researchers. Any discrepancies identified during the verification process were resolved through discussion or, when necessary, by consultation with a third reviewer. Throughout the extraction process, strict adherence to predefined extraction criteria was maintained to ensure consistency. All extraction procedures and decisions were thoroughly documented to allow for review and verification.

### 2.4 Risk of bias and certainty of evidence

To ensure rigorous evaluation the risk of bias, two independent investigators conducted assessment using the Cochrane Risk of Bias Tool (RoB 2.0), with any disagreements resolved through consensus or adjudication by a third reviewer. And the assessment results were reported in Supplementary Figures S1, S2.

To assess heterogeneity among included studies, Chi-square test and I^2^ statistic was both employed. If *p* < 0.10, it indicates statistically significant heterogeneity, and the actual degree of variability should be interpreted in conjunction with the I^2^ value. Publication bias was evaluated through funnel plot asymmetry testing. Moreover, subgroup analyses was performed to investigate potential sources of variation.

### 2.5 Statistical analysis

For dichotomous outcomes, event counts and total sample sizes were extracted. Continuous outcomes required means, standard deviations, and participant numbers for both groups. For studies reporting medians and interquartile ranges instead of means and standard deviations, we performed conversions using validated methods (via the estmeansd web tool: https://smcgrath.shinyapps.io/estmeansd/).

Statistical analyses were conducted using Review Manager 5 (RevMan 5, The Cochrane Collaboration), with results presented as forest plots illustrating pooled effect estimates and their corresponding 95% confidence intervals. To assess the robustness of the findings, trial sequential analysis (TSA) was performed to determine whether the cumulative sample size was sufficient to reach conclusive results.

## 3 Results

### 3.1 Screening

Total of 408 searches were identified from systematic literature search. After screening, 13 RCTs were enrolled. Additionally, 4 RCTs were identified through manual searching, resulting in 17 studies (*N* = 1,422 patients) eventually included as show in Figure 1.

### 3.2 Characteristics of included RCTs

The characteristics of the included studies were presented in Table 1. Based on our predefined criteria, 23.5% (4/17) of the studies were multicenter trials, with the remaining being single-center studies. Across all trials, the mean/median age ranged from 40.2 to 72.1 years, with male patients comprising 53.8% (595/1,101) of participants (gender was unspecified for 321 patients in 5 studies). Disease severity assessments showed that 16 trials reported APACHE II scores ranging from 11.1 to 38, while 11 studies documented SOFA scores between 4 and 12.4.

Among the included studies, 8 trials (586/1,422 patients, 41.2%) compared DEX with PROP. However, three of them used PROP in combination with other sedation as the control group. To ensure the validity of comparative results, only 5 studies (representing 29.4% [419/1,422] of patients) were ultimately included in the DEX vs. PROP subgroup analysis.

### 3.3 Risk of bias

**Figure 2:**
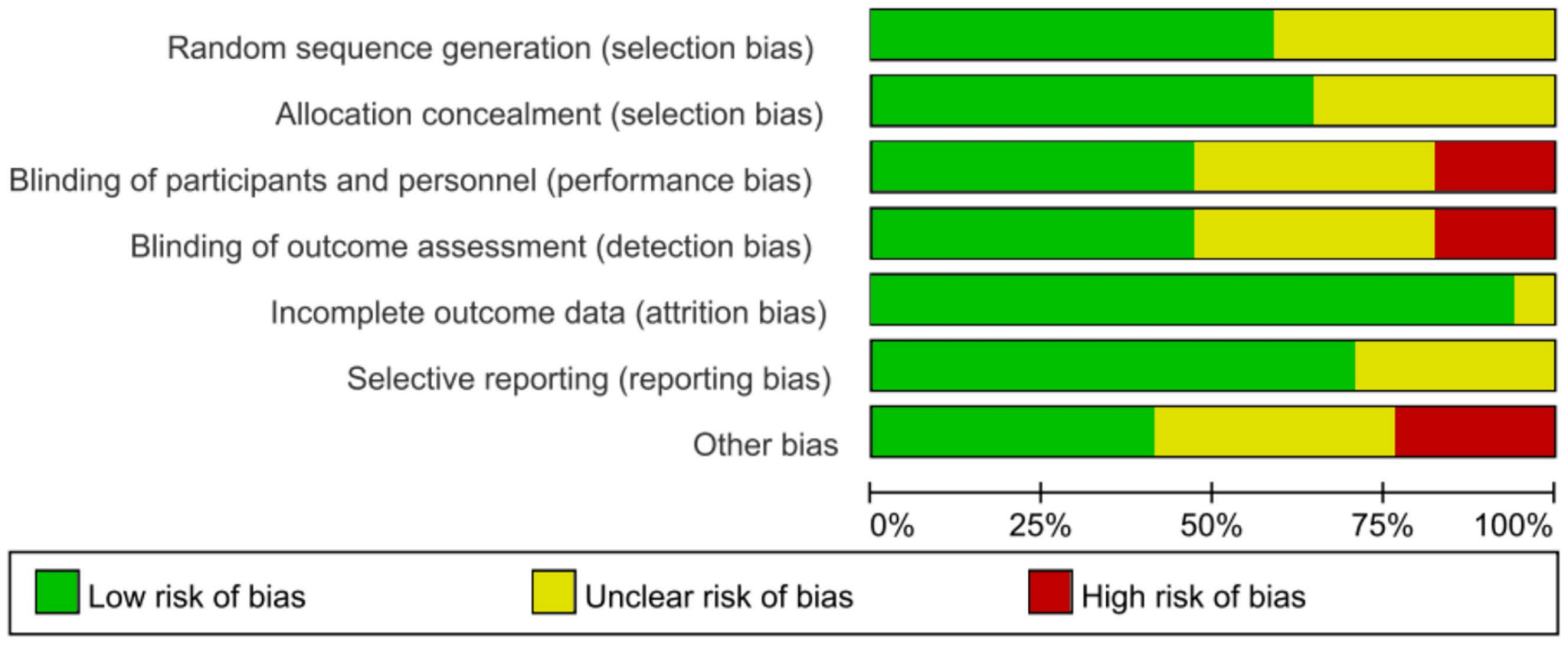
Risk of bias (RoB) summary graph.

Risk of bias for all included studies was assessed by the Cochrane RoB 2.0, and the methodological quality assessments were summarized in Figure 2, with full details provided in Supplementary Figure S1. Over half of the studies (53%) described appropriate random sequence generation and allocation concealment, and were judged as low risk. However, 8 studies failed to report allocation concealment methods, raising some concerns. Blinding of performance/detection was adequately performed in 8 studies, while 3 studies had high risk due to open-label design and 6 studies did not report blinding methods. Most studies (16 studies) with attrition rates <10%, and 12 studies had a low risk of reporting bias by comparing the study protocol (clinical trial registry records or pre-specified primary/secondary outcomes) with the final reported outcome measures. In addition, these studies may also be subject to other sources of bias, such as baseline imbalance, early trial stopping, and adherence bias as mentioned above, etc.

### 3.4 Duration of IMV

**Figure 3:**
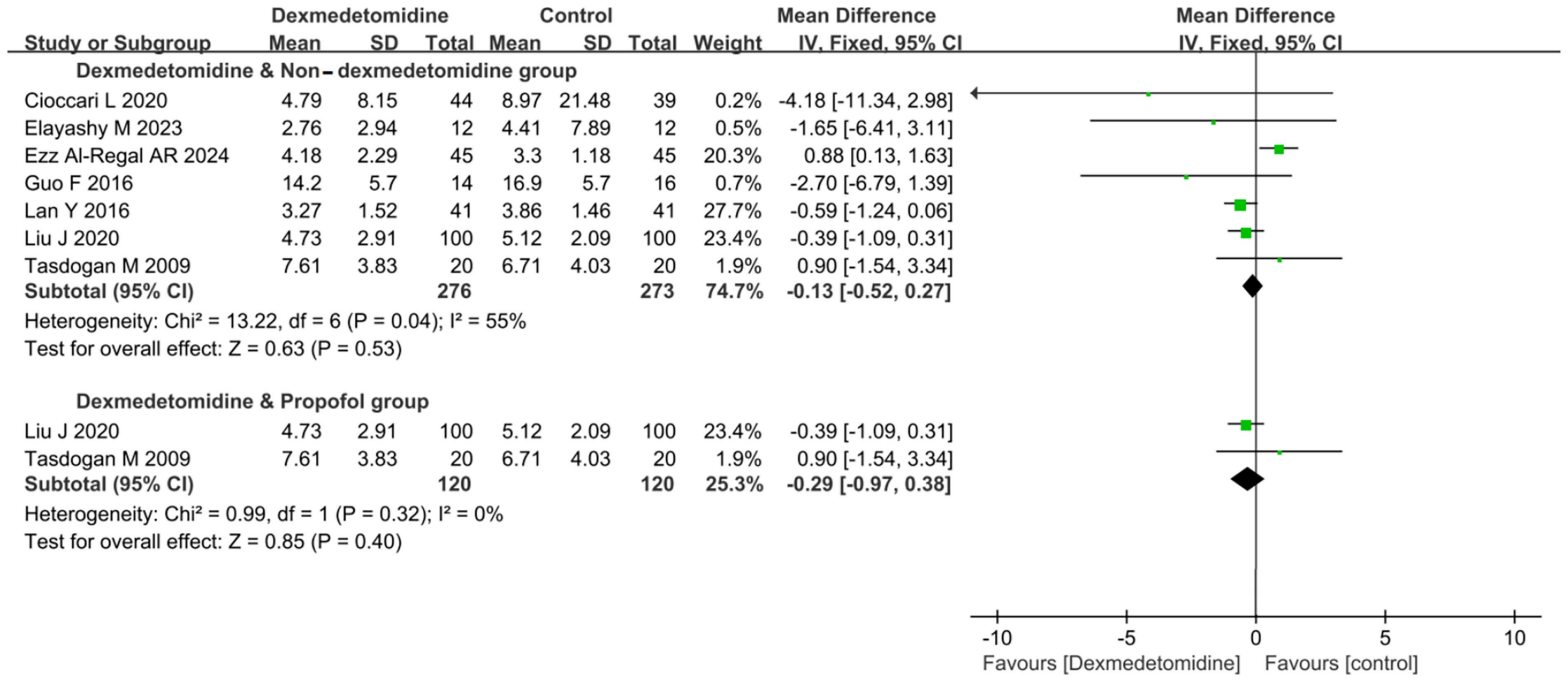
Forest plot of duration of invasive mechanical ventilation (IMV). All outcome data for the duration of IMV were presented as mean ± standard deviation (SD) with the unit of measurement in days. The results were presented as forest plots illustrating pooled effect estimates and their corresponding 95% confidence intervals (CI).

7 RCTs (involving 549 patients) evaluated the impact of DEX on the duration of IMV. Meta-analysis revealed no significant difference in IMV duration between patients receiving DEX and non-DEX (mean difference [MD] − 0.13 days, 95% CI − 0.52 to 0.27, *p* = 0.53; I^2^ = 55%, *p* = 0.040; Figure 3; Supplementary Figure S2).

To further clarify the effects of DEX and PROP on the duration of IMV, a subgroup analysis was conducted. Nevertheless, it did not reveal a statistically significant difference in IMV duration between the DEX and PROP groups (MD − 0.29 days, 95% CI − 0.97 to 0.38, *p* = 0.40; I^2^ = 0%, *p* = 0.32; Figure 3; Supplementary Figure S2).

### 3.5 Duration of ICU LOS

**Figure 4:**
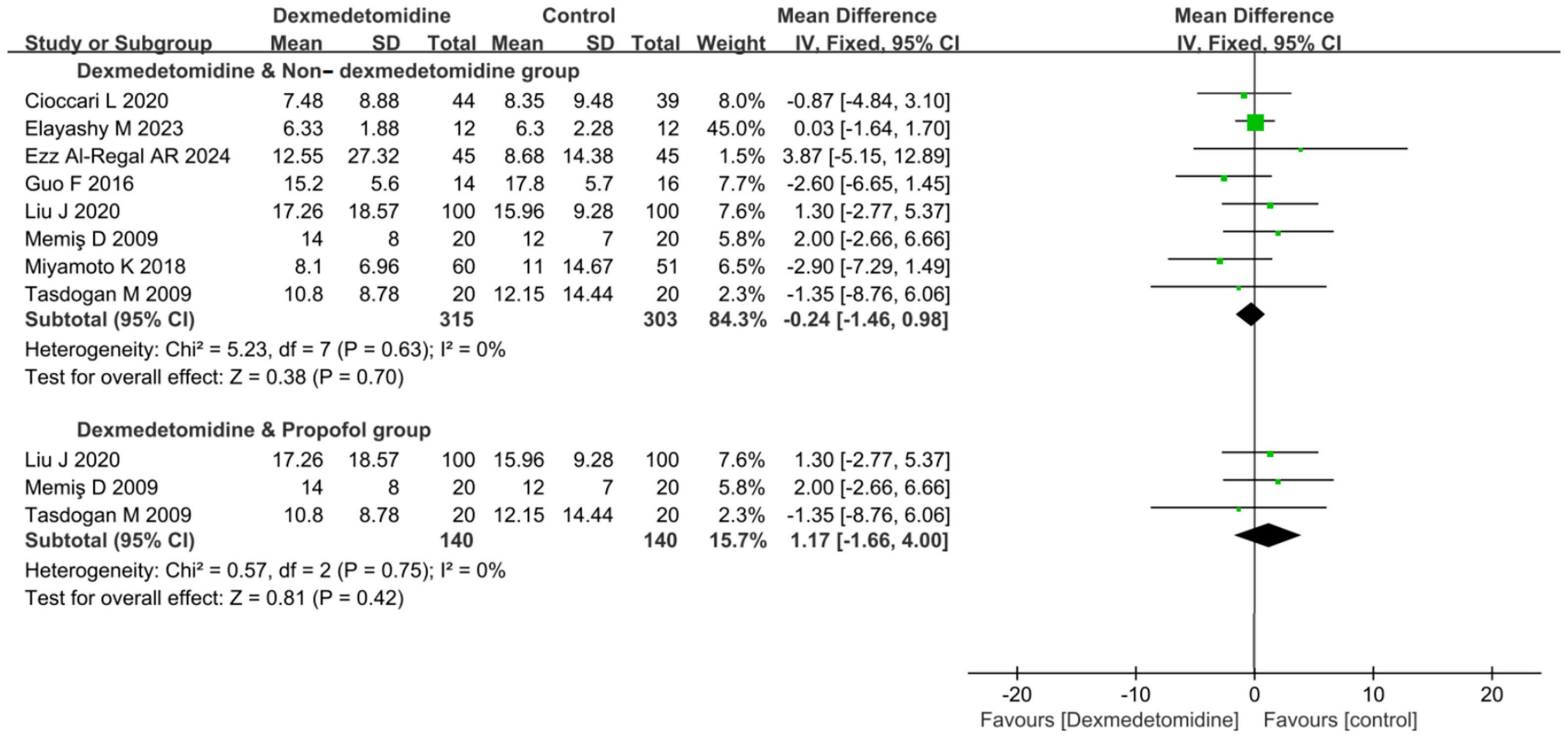
Forest plot about duration of ICU LOS. All outcome data for the duration of IMV were presented as mean ± standard deviation (SD) with the unit of measurement in days. The results were presented as forest plots illustrating pooled effect estimates and their corresponding 95% confidence intervals (CI). ICU LOS, Length of stay in intensive care unit.

8 RCTs (comprising a total of 618 patients) reported data about ICU LOS. But, the pooled result showed that DEX administration was not associated with a statistically significant reduction in ICU LOS compared to control groups not receiving DEX (MD −0.24 days, 95% CI -1.46 to 0.98, *p* = 0.70; I^2^ = 0%, *p* = 0.63; Figure 4; Supplementary Figure S3).

Subgroup analysis comparing DEX specifically against PROP revealed no significant difference in ICU LOS yet (MD 1.17 days, 95% CI -1.16 to 4.00, *p* = 0.42; I^2^ = 0%, *p* = 0.75; Figure 4).

### 3.6 28-day mortality

**Figure 5:**
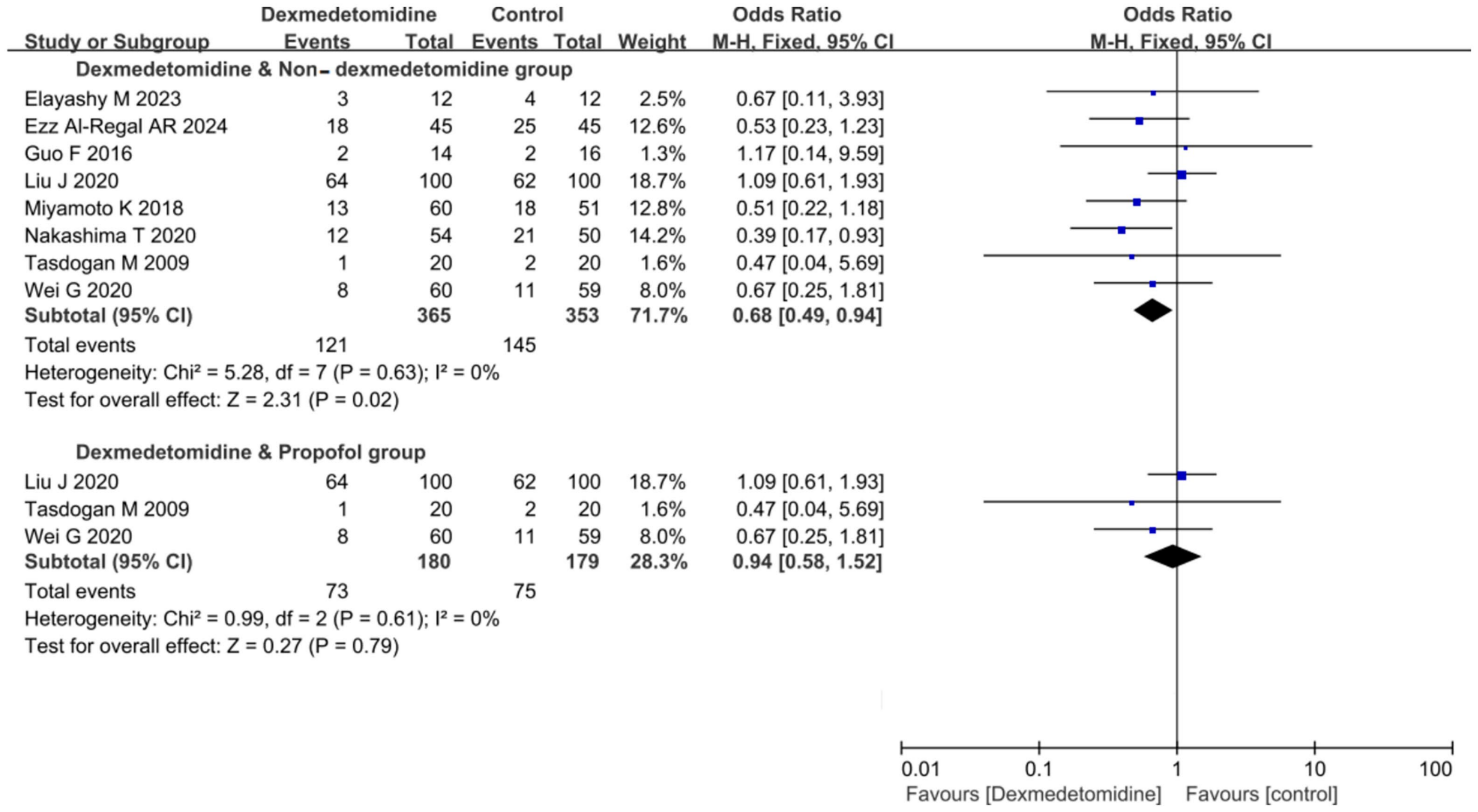
Forest plot of 28-day mortality. The results were presented as forest plots illustrating pooled effect estimates and their corresponding 95% confidence intervals (CI).

**Figure 6:**
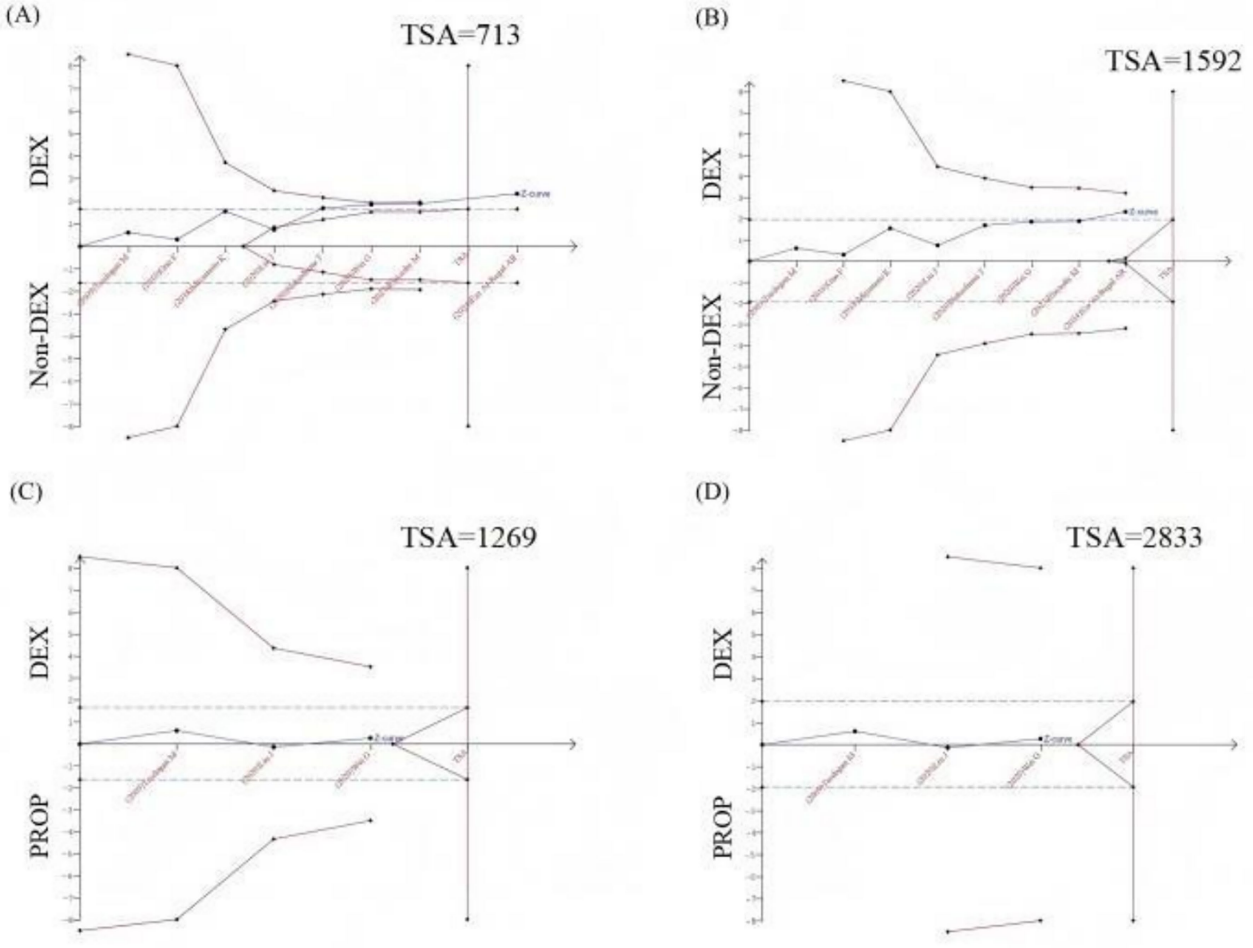
Trial sequential analysis (TSA) of 28-day mortality. **(A)** TSA of 28-day mortality comparing DEX vs. non-DEX (Type I Error: 10.0%, Power: 70.0%); **(B)** TSA of 28-day mortality comparing DEX vs. non-DEX (type I error: 5.0%, Power: 90.0%); **(C)** TSA of 28-day mortality comparing DEX vs. PROP (Type I Error: 10.0%, Power: 70.0%, RRR: 12.5%); **(D)** TSA of 28-day mortality comparing DEX vs. PROP (Type I Error: 5.0%, Power: 90.0%, RRR: 12.5%); X-axis: represented the cumulative sample size in chronological order of publication; Y-axis: represented the Z-score; The blue curve was the “Z-curve,” indicating the trend of cumulative Z-scores as the number of sample size increases. The blue dashed line denoted the conventional boundary, while the red curve represents the TSA boundary. The red vertical line indicated the required sample size to achieve a stable conclusion. DEX, Dexmedetomidine; PROP, Propofol; RRR, Relative risk reduction.

8 RCTs (comprising 718 patients) evaluated 28-day mortality outcomes. The meta-analysis demonstrated that DEX administration was associated with a statistically significant reduction in 28-day mortality compared to control groups not receiving DEX (odds ratio [OR] 0.68, 95% CI 0.49 to 0.94, *p* = 0.02; I^2^ = 0%, *p* = 0.63; Figure 5; Supplementary Figure S4). The TSA of 28-day mortality of DEX compared to control groups (non-DEX) also confirmed that the current sample size of the studies could yield stable conclusions under the conditions of Type 1 Error: 10.0%, Power: 70.0%, and the current cumulative effect type intervention (mortality rate in the DEX group: 33.15%; mortality rate in the non-DEX group: 41.1%; Figure 6A). However, if the goal was to further reduce the Type 1 Error rate to 5% and increase the power to 90% while maintaining the current cumulative effect type intervention, the sample size would need to be expanded to 1,592 (Figure 6B).

The lower 28-day mortality in the DEX group compared to the non-DEX group may attribute to DEX’s significant reduction in inflammatory markers compared to non-DEX groups, including IL-6 (MD −3.11 ng/L, 95% CI −5.32 to −0.90, *p* = 0.006; I^2^ = 97%, *p* < 0.001; Supplementary Figures S5A,C) and TNF-*α* (MD −0.21 ng/L, 95% CI −0.30 to −0.12, *p* < 0.001; I^2^ = 99%, *p* < 0.001; Supplementary Figures S5B,D). Importantly, the incidence of adverse effects did not increase compared to non-DEX groups, as evidenced by delirium (OR 0.82, 95% CI 0.34 to 1.97, *p* = 0.66; I^2^ = 56%, *p* = 0.10; Supplementary Figures S6A,B), bradycardia (OR 1.36, 95% CI 0.66 to 2.78, *p* = 0.40; I^2^ = 61%, *p* = 0.05; Supplementary Figures S6C,D), and hypotension (OR 1.38, 95% CI 0.59 to 3.19, *p* = 0.46; I^2^ = 62%, *p* = 0.07; Supplementary Figures S6E,F). However, the substantial heterogeneity observed in these results (I^2^ > 50% for most analyses) suggests the need for further studies to enhance the robustness of these findings.

In the subgroup analysis comparing DEX specifically with PROP, no significant difference in 28-day mortality was observed (OR 0.94, 95% CI 0.58 to 1.52, *p* = 0.79; I^2^ = 0%, *p* = 0.61; Figure 5; Supplementary Figure S4). The currently available RCTs comparing DEX and PROP that reported 28-day mortality were limited in number and statistical power, precluding definitive conclusions about their treatment effects. Based on the existing evidence, we hypothesized a relative risk reduction (RRR) of 12.5%. Trial sequential analysis (TSA) of 28-day mortality comparing DEX and PROP demonstrated that the current sample size was insufficient to reach conclusive findings. Under the prespecified parameters (*α* = 0.10, power = 70%), a minimum of 1,269 participants would be required to achieve robust conclusions (Figure 6C). However, to achieve a more stringent type I error rate of 5% with 90% statistical power, the required sample size would increase substantially to 2,833 participants (Figure 6D).

Regrettably, the available comparative studies between DEX and PROP provided limited relevant data, which precluded meta-analysis of their effects on inflammatory markers and adverse events as discussed above.

## 4 Discussion

In our study, DEX demonstrated the advantage of improving 28-day mortality of patients with septic shock, and the incidence of adverse events did not increase. A meta-analysis of 19 RCTs (1,929 patients) reached conclusions consistent with our results, showing that compared with benzodiazepines, DEX could significantly reduce mortality (14). But, it was not entirely consistent with previous conclusions derived from all septic patients, including shock cases. A meta-analysis of 10 RCTs demonstrated that in mechanically ventilated septic patients, DEX was associated with reduced ICU LOS, but did not significantly affect 28-day mortality, hospital mortality, or ventilator-free days (15). Compared to the non-shock sepsis population, septic shock patients exhibit distinct disease pathophysiology and hemodynamic characteristics that confer unique patterns of sedation effects on clinical outcomes. Therefore, it required a dedicated analyzed—which was the primary rationale for conducting this meta-analysis.

The observed mortality reduction may be attributable to the following potential mechanisms: DEX had a de-catecholaminization effect, which can alleviate tachycardia in septic shock caused by hypovolemia, vascular paralysis, fever, and adrenergic hyperactivity. This persistent tachycardia was associated with higher mortality in sepsis. Clinical studies based on speckle-tracking echocardiography confirmed that DEX combined with a bundle strategy could stabilize the heart rate in patients with severe sepsis, reduce myocardial oxygen consumption, and effectively shorten the recovery time, improving the overall prognosis (16).

However, in the specific population of septic shock patients, DEX demonstrated no significant difference in clinical outcomes compared to PROP in our study, including: 28-day mortality, duration of IMV and ICU LOS. But, a meta-analysis including 7 studies (a total of 1,212 patients) comparing DEX with PROP reported that administration of DEX for sedation in septic patients could shorten ICU LOS (17). Although our analysis showed limited heterogeneity, the findings should be interpreted cautiously given the small number of included studies and insufficient sample size (below the TSA-estimated requirement). And, future studies should include more targeted RCTs to derive more robust conclusions.

Due to the limitation of extractable data from current literature, it could not conclusively determine whether DEX and PROP induce differential hemodynamic effects, while clinical concerns exist regarding DEX use in septic shock patients—primarily due to its vasodilatory effects (potentially causing hypotension) and α2-receptor-mediated sinus node dysfunction with significant bradycardia. A prior multicenter RCT in septic patients demonstrated comparable frequencies of adverse hemodynamic events (34.4% vs. 16.1%, *p* = 0.065) but significantly more profound hypotension with propofol (mean blood pressure reduction: 47.3 vs. 34.7 mmHg, *p* = 0.031), suggesting potential clinically relevant differences in hemodynamic profiles between these sedatives that warrant further investigation in adequately powered prospective studies (18).

Current evidence suggests that de-catecholaminization may not universally benefit all sepsis patients, particularly given the heterogeneous cardiovascular phenotypes observed in septic shock (19). The strategy of pharmacologically reducing heart rate in compensatory tachycardia could adversely affect cardiac output. Contrary to expectations, DEX did not exacerbate hemodynamic instability in the critical phase of septic shock, showing comparable rates of hypotension and bradycardia to other sedative-analgesics. This phenomenon may be explained by DEX’s demonstrated ability to improve microcirculatory alterations during initial septic shock resuscitation (20). Furthermore, existing literature confirms that while DEX may increase arrhythmia incidence compared to other sedatives in sepsis patients, it shows no significant difference in overall adverse event rates (14). The drug’s potential cardioprotective benefits are multifaceted, including modulation of programmed cell death pathways, autophagy regulation, anti-fibrotic effects, inflammation reduction, and improvements in endothelial function, microcirculation, mitochondrial performance, hemodynamic stability, and arrhythmia management (21). These complex mechanisms underscore the need for phenotype-specific therapeutic approaches in sepsis management.

Additionally, animal studies found that DEX could also reduce mortality in rats with toxic shock by regulating inflammation and autophagy-related signaling pathways (22, 23). Previous evidence-based medical studies showed that compared to other sedatives, DEX could significantly reduce the inflammatory response in patients with sepsis (14). Our analysis results also demonstrated this advantage in the stage of septic shock, as the levels of IL-6 and TNFα in the DEX group were significantly lower than those in non-DEX group. However, due to the high heterogeneity of the included studies, the evidence remained limited. Potential contributing factors to these findings beyond the limitations of available study data may include that patients with septic shock were in the stage of cytokine storm, where inflammatory factors themselves fluctuate greatly. Additionally, there was heterogeneity in the time points at which inflammatory factors were monitored in the included studies, and there are differences in the sedation strategies used in the control group, all of which can affect the results of the comparison.

At the same time, compared to other sedatives, the incidence of delirium in patients with septic shock treated with DEX for sedation did not significantly decrease. These results demonstrated partial inconsistency with current evidence, necessitating focused consideration in clinical interpretation. The infusion rate could affect the incidence of delirium (24), which in turn could directly impact patient prognosis (25, 26). The current evidence could not conclusively establish a causal relationship with administration speed and total drug dosage due to dataset constraints, necessitating prospective studies to verify this postulated mechanism.

This study had the following limitations regarding the reliability of results: First, while the inclusion of RCTs exclusively strengthened the evidence quality, it was possible that some clinically meaningful findings from non-RCTs were overlooked. Moreover, despite efforts to minimize bias, the selective incorporation of data from multi-group comparisons beyond our study design may introduce selection bias by overlooking the broader trial context. Second, incomplete reporting of key data in some studies resulted in certain data being excluded from the analysis or requiring estimation methods, which may affect the accuracy of the results. And, while the odds ratio (OR) offers computational stability with zero-cell counts in dichotomous outcomes (particularly for adverse events), it may overestimate the effect size compared to the risk ratio (RR), especially for common outcomes. Additionally, some analyses demonstrated a certain degree of heterogeneity. Although we attempted to explore the sources of heterogeneity through subgroup analysis, the limited number of included studies prevented a comprehensive assessment of all potential influencing factors. Forth, the extended time span of the included studies may have led to reduced comparability between studies from different periods due to evolving diagnostic and treatment standards. Fifth, despite systematically searching multiple databases, there remains a possibility of missing unpublished studies or non-English literature. Finally, regarding the applicability of results, the studies included in this research primarily originated from Upper-middle-income countries and specific populations (e.g., adult patients). This limitation may affect the generalizability of the conclusions to other regions and populations. Furthermore, differences between the standardized protocols used in the studies and actual clinical practice (27, 28) should also be carefully considered when interpreting the results.

## 5 Conclusion

Based on the analysis of existing RCTs, DEX demonstrated superiority over non-DEX sedatives for sedation in critically ill patients with septic shock, particularly in improving 28-day mortality, without increasing hemodynamic adverse events. However, current evidence showed no significant differences in primary clinical outcomes between DEX and PROP, warranting further high-quality RCTs for definitive conclusions.

## Data Availability

The data analyzed in this study is subject to the following licenses/restrictions: the datasets used and/or analyzed during the present study are available from the corresponding author on reasonable request. Requests to access these datasets should be directed to Wenjiao Wang, wangwenjiao_tj3zx@163.com.
