# Supplementary material for: Is dexmedetomidine superior to non-dexmedetomidine sedatives (particularly propofol) for sedation in critically ill patients with septic shock? A systematic review and meta-analysis of randomized controlled trials
Source: Front Med (Lausanne). 2025 Oct 9;12:1646256. doi: 10.3389/fmed.2025.1646256 (PMC12546090; doi:10.3389/fmed.2025.1646256)
Supplement: Supplementary file 2 [file Data_Sheet_2.docx]

## **Supplementary Material 2: Searching strategies**

| **Database** | **Search strategy** | | | **References identified** |
| --- | --- | --- | --- | --- |
| CENTRAL | MeSH | #1 | MeSH descriptor: [Shock, Septic] explode all trees | 1450 |
|  | MeSH | #2 | MeSH descriptor: [Dexmedetomidine] explode all trees | 3115 |
|  | Free text search | #3 | (“septic shock” and “dexmedetomidine”):ti,ab,kw (Word variations have been searched) | 43 |
|  | Total | #4 | (#1 and #2) or #3 | 43 |
| EMBASE | Emtree | #1 | 'sepsis-associated hypotension'/exp OR 'sepsis-associated hypotension' OR 'septicaemic shock'/exp OR 'septicaemic shock' OR 'septicemic shock'/exp OR 'septicemic shock' OR 'shock, septic'/exp OR 'shock, septic' OR 'septic shock'/exp OR 'septic shock' | 87,479 |
|  | Emtree | #2 | 'bxcl 501'/exp OR 'bxcl 501' OR 'bxcl501'/exp OR 'bxcl501' OR 'cepedex'/exp OR 'cepedex' OR 'da 9501'/exp OR 'da 9501' OR 'da9501'/exp OR 'da9501' OR 'delos'/exp OR 'delos' OR 'dexamedetomidine'/exp OR 'dexamedetomidine' OR 'dexdomitor'/exp OR 'dexdomitor' OR 'dexdor'/exp OR 'dexdor' OR 'dexmedetomidine hydrochloride'/exp OR 'dexmedetomidine hydrochloride' OR 'igalmi'/exp OR 'igalmi' OR 'mpv 1440'/exp OR 'mpv 1440' OR 'mpv1440'/exp OR 'mpv1440' OR 'precedex'/exp OR 'precedex' OR 'primadex'/exp OR 'primadex' OR 'sedadex'/exp OR 'sedadex' OR 'sileo'/exp OR 'sileo' OR 'tpu 006'/exp OR 'tpu 006' OR 'tpu006'/exp OR 'tpu006' OR 'dexmedetomidine'/exp OR 'dexmedetomidine' | 24,512 |
|  | Emtree | #3 | #1 AND #2 | 242 |
|  | Free text search | #4 | ('septic shock'/exp OR 'septic shock') AND ('dexmedetomidine'/exp OR 'dexmedetomidine') | 237 |
|  | Total | #5 | #3 OR #4 | 242 |
| WOS | Advanced research | / | Refine results for "septic shock" (Title) AND "septic shock" (Abstract) AND "dexmedetomidine" (Topic) AND "dexmedetomidine" (Abstract) and Preprint Citation Index (Exclude-Database) | 27 |
| Pubmed | MeSH | #1 | "Septic Shock"[Title/Abstract] OR "shock endotoxic"[Title/Abstract] OR "Endotoxin Shock"[Title/Abstract] OR "Endotoxin Shocks"[Title/Abstract] OR "shock endotoxin"[Title/Abstract] OR "shock toxic"[Title/Abstract] OR "Toxic Shock"[Title/Abstract] OR "Toxic Shock Syndrome"[Title/Abstract] OR "shock syndrome toxic"[Title/Abstract] OR "Toxic Shock Syndromes"[Title/Abstract] | 37,091 |
|  | MeSH | #2 | "Dexmedetomidine Hydrochloride"[Title/Abstract] OR "hydrochloride dexmedetomidine"[Title/Abstract] OR "mpv 1440"[Title/Abstract] OR "MPV1440"[Title/Abstract] OR "mpv 1440"[Title/Abstract] OR "Precedex"[Title/Abstract] OR "Igalmi"[Title/Abstract] OR "Sileo"[Title/Abstract] OR "Dexdor"[Title/Abstract] OR "Dexdomitor"[Title/Abstract] | 167 |
|  | Free text search | #3 | "septic shock"[Title/Abstract] AND "dexmedetomidine"[Title/Abstract] | 49 |
|  | Total | #3 or (#1 and #2) | 3[UID] OR (("Septic Shock"[Title/Abstract] OR "shock endotoxic"[Title/Abstract] OR "Endotoxin Shock"[Title/Abstract] OR "Endotoxin Shocks"[Title/Abstract] OR "shock endotoxin"[Title/Abstract] OR "shock toxic"[Title/Abstract] OR "Toxic Shock"[Title/Abstract] OR "Toxic Shock Syndrome"[Title/Abstract] OR "shock syndrome toxic"[Title/Abstract] OR "Toxic Shock Syndromes"[Title/Abstract]) AND ("Dexmedetomidine Hydrochloride"[Title/Abstract] OR "hydrochloride dexmedetomidine"[Title/Abstract] OR "mpv 1440"[Title/Abstract] OR "MPV1440"[Title/Abstract] OR "mpv 1440"[Title/Abstract] OR "Precedex"[Title/Abstract] OR "Igalmi"[Title/Abstract] OR "Sileo"[Title/Abstract] OR "Dexdor"[Title/Abstract] OR "Dexdomitor"[Title/Abstract])) | 50 |
| VIP | Advanced research | / | 篇关摘=脓毒性休克 AND 篇关摘=右美托咪定 | 24( in Chinese)/67 (in English) |
| CNKI | Advanced research | / | （篇关摘：脓毒性休克(精确)）AND（篇关摘：右美托咪定(精确)） | 22 |
| **Total** | | | | **408** |

CENTRAL, Cochrane Central Register of Controlled Trials; EMBASE, Excerpta Medica Database; WOS, Web of Science; VIP, China Science and Technology Journal Database; CNKI, China National Knowledge Infrastructure;
