# Supplementary material for: Is dexmedetomidine superior to non-dexmedetomidine sedatives (particularly propofol) for sedation in critically ill patients with septic shock? A systematic review and meta-analysis of randomized controlled trials
Source: Front Med (Lausanne). 2025 Oct 9;12:1646256. doi: 10.3389/fmed.2025.1646256 (PMC12546090; doi:10.3389/fmed.2025.1646256)
Supplement: Supplementary file 3 [file Supplementary_file_1.docx]

# Supplementary Figures


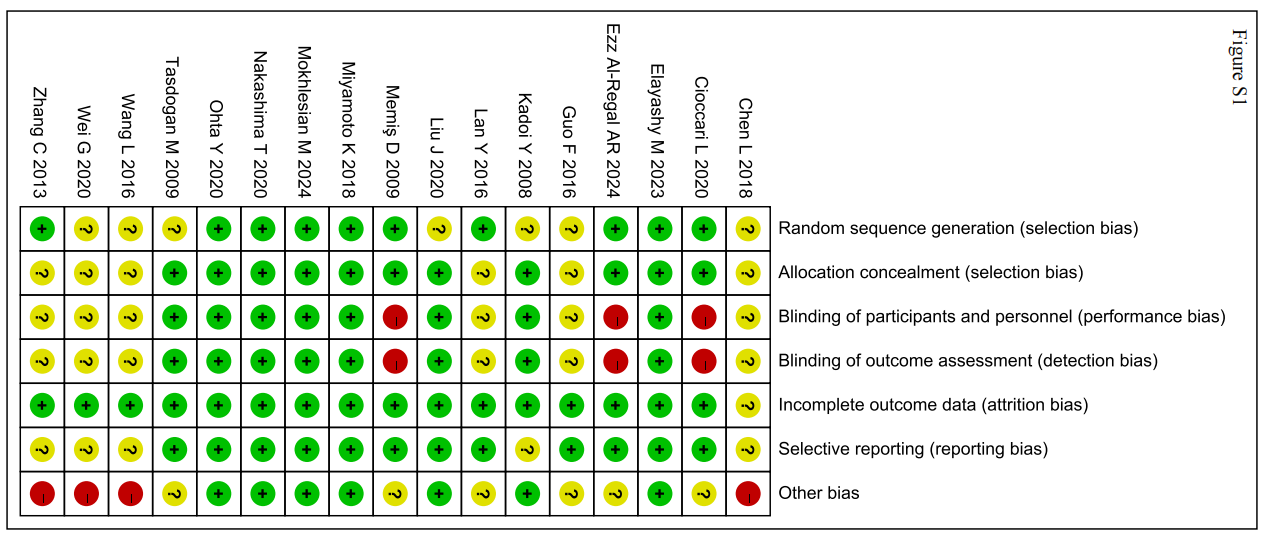


**Figure S1 Risk of bias assessment for individual studies.**Green indicated low risk of bias, yellow indicated unclear risk of bias, and red indicated high risk of bias.


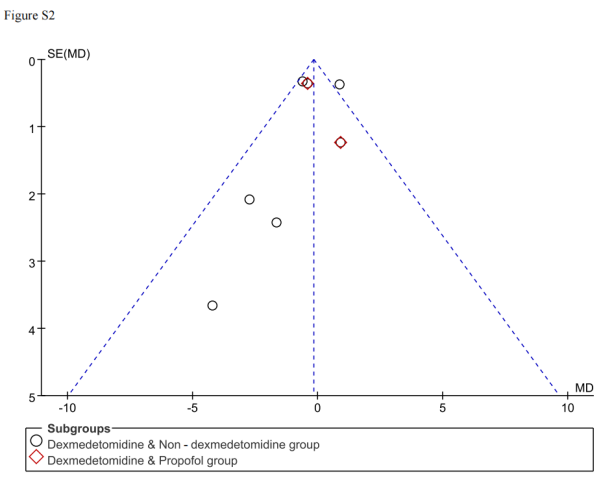


**Figure S2** **Funnel plot analyzing publication bias in duration of invasive mechanical ventilation (IMV).**


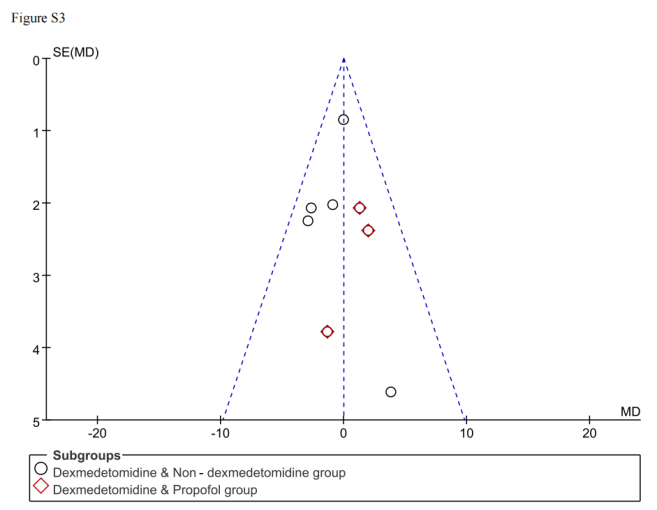


**Figure S3** **Funnel plot analyzing publication bias in duration of ICU LOS.** ICU LOS: Length of stay in intensive care unit;


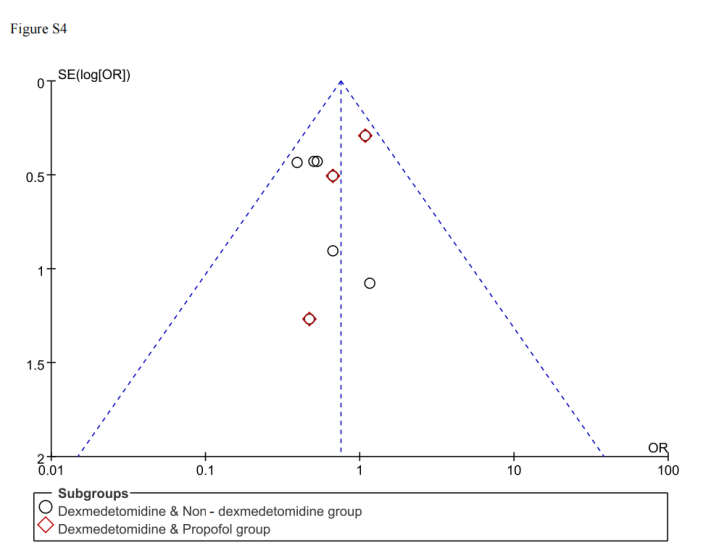


**Figure S4** **Funnel plot analyzing publication bias in 28-day mortality.**


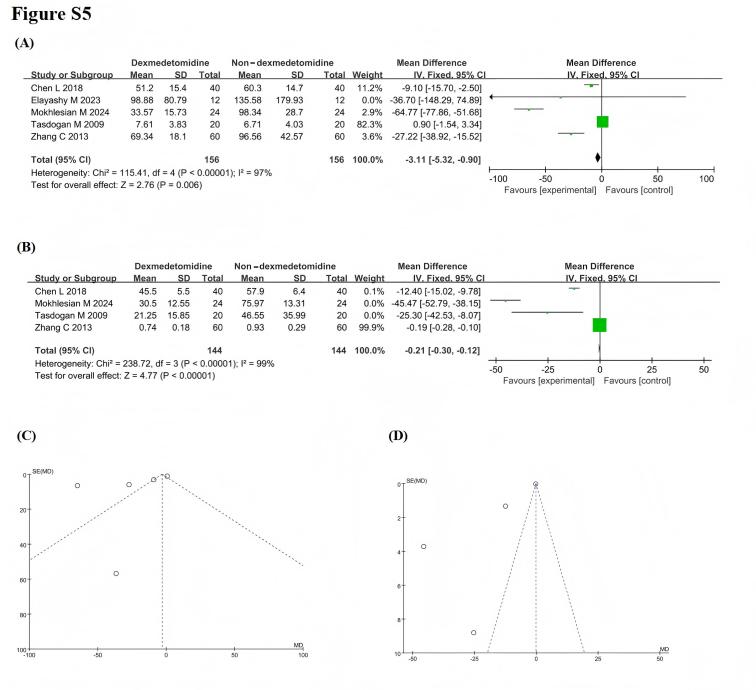


**Figure S5 Meta-analysis results comparing inflammatory factors between dexmedetomidine (DEX) and non-DEX.** (A) and (B) were forest Plot of interleukin-6 (IL-6) and tumor necrosis factor-alpha (TNF-α). Data was presented as mean ± standard deviation (SD) with the unit of measurement in ng/L, and results were presented as forest plots illustrating pooled effect estimates and their corresponding 95% confidence intervals (CI). (C) and (D) were funnel plot analyzing publication bias in IL-6 and TNF-α.


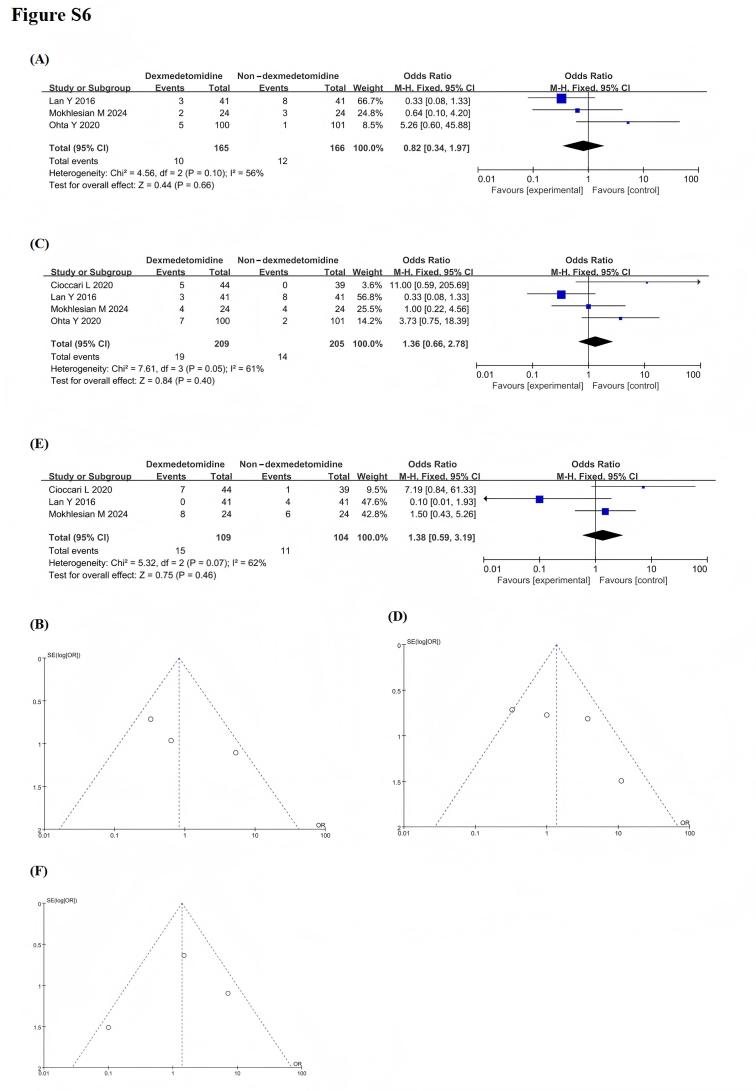


**Figure S6 Results of the meta-analysis on the incidence of adverse events between dexmedetomidine (DEX) and non-DEX.** (A), (C) and (E) were the forest plot of delirium, bradycardia and hypotension. The results were presented as forest plots illustrating pooled effect estimates and their corresponding 95% confidence intervals (CI). (B), (D) and (F) were funnel plot analyzing publication bias in delirium, bradycardia and hypotension.
